# Mortality attributable to tobacco: review of different methods

**DOI:** 10.1186/2049-3258-72-22

**Published:** 2014-07-01

**Authors:** Nabil Tachfouti, Chantal Raherison, Majdouline Obtel, Chakib Nejjari

**Affiliations:** 1 University of Bordeaux, Bordeaux, France; 2 Department of Respiratory Diseases, CHU Bordeaux, France; 3 Department of Epidemiology and Disease control, Ministry of Health, Rabat, Morocco; 4 Laboratory of Epidemiology, Clinical Research and Community Health -Faculty of Medicine, KM: 2.2 Route de Sidi Harazem, Fez, Morocco

**Keywords:** Tobacco, Attributable risk, Mortality, Modelization

## Abstract

**Background:**

One of the most important measures for ascertaining the impact of tobacco is the estimation of the mortality attributable to its use. Several indirect methods of quantification are available. The objective of the article is to assess methodologies published and applied in calculating mortality attributable to smoking.

**Methods:**

A review of the literature was made for the period 1998 to 2005, in the electronic databases MEDLINE. Twelve articles were selected for analysis.

**Results:**

The most widely used methods were the prevalence methods, followed by smoking impact ration method. Ezzati and Lopez showed that the general rate of Smoking attributable mortality (SAM) globally was 12% (18% in men). Across countries, attributable fractions of total adult deaths ranged from 8% in Southern Africa, 13.6% in Brazil (18.1% in men) and 25% in Hong Kong (33% in men).

**Conclusion:**

The variations can be attributed to methodological differences and to different estimates of the main tobacco-related illnesses and tobacco prevalence. All methods show limitations of one type or another, yet there is no consensus as to which furnishes the best information.

## Background

Non-communicable diseases are rapidly increasing in many developing countries, largely due to demographic and lifestyle changes. It is estimated that nearly half the disease burden in low and middle-income countries (LMIC) is from non-communicable diseases, and more than 21% of deaths in such countries are due to cardiovascular diseases [1]. Globally, many of the risk factors for heart disease, diabetes, cancer and pulmonary diseases are due to lifestyle and can be prevented.

Among risk factors for non-communicable diseases, tobacco is enemy number one [2]. It is a widely established cause of cancer, and moreover, also responsible for cardiovascular and chronic respiratory diseases. The growth in smoking rates is followed ten to twenty years later by the increase in the incidence of diseases such as ischemic heart disease, lung, oral cavity and larynx cancers; and 20 to 40 years later, by chronic obstructive airway disease [3,4]. Tobacco impact on society can be measured in terms of the mortality burden, which represents tobacco-attributable deaths [5].

It has been estimated that there are more than 1.3 billion smokers worldwide, with around 82% residing in LMIC [6]. Eighty five percent of the world’s population lives in LMIC [7]. 10% of all deaths resulting from non-communicable diseases (including cancer, cardiovascular, chronic respiratory diseases, and diabetes) are related to tobacco and most of these occur in these countries [8].

In people over age 30, smoking accounts for one in every five deaths among men and one in every 20 deaths among women globally [9]. The World Health Organization (WHO) has estimated that approximately 5.4 million people died worldwide from tobacco-related illnesses in 2006 [10]. Unless urgent action is taken, tobacco’s annual death toll will rise to more than eight million by the year 2030. More than 80% of those deaths will be in LMIC [10]. Over five million of these deaths are attributed directly to smoking, and about 600,000 to second hand smoke [11], people who they do not smoke but breathe air polluted by poisonous gases from those who smoke. It is estimated that in the period 2002/2030, tobacco-attributable deaths will decrease by 9% in developed countries, but increase by 100% (to 6.8 million) in developing countries [12].

Ezzati et al. [13] estimated that 11% of all cardiovascular deaths in the world in 2000 could be attributed to tobacco, in particular ischemic heart disease and cerebrovascular disease [14]. In addition, cancer has been attributed to 21% of all cancer deaths in the world, including 29% of deaths in developed countries and 18% in developing countries [15]. The UK prospective study of smoking and death among British doctors that began in 1951 found that men born in 1900–1930 who smoked only cigarettes and continued smoking died on average about 10 years younger than lifelong non-smokers. Cessation at age 60, 50, 40, or 30 years gained, respectively, about 3, 6, 9, or 10 years of life expectancy [16].

Although the negative effects of smoking on mortality at the individual level are well established, measuring the mortality impact at the population level is more challenging because of the difficulty of obtaining accurate cohort histories of smoking behavior. Cohort data are necessary because smoking generally begins relatively early in life, whereas the full impact on mortality is not revealed until at much older ages [17]. The most persuasive evidence identifying the mortality risks associated with smoking has been drawn from prospective cohort studies that compare the death rates of current smokers and former smokers with the death rates of those who never smoked regularly. The Cancer Prevention Study II (CPS-II) in the USA is the largest such study, but is based on a sample of volunteers who are more likely to be White, middle class and college-educated than the US population as a whole [18].

Smoking attributable mortality (SAM) has been widely used in studies and is considered to be one of the most relevant summary statistics. Due to its capacity to show the harm that tobacco causes to health, it would be of help in the planning of health policy. The task of quantifying SAM has been performed mainly through indirect methods. This review sought to describe the different methods of estimating mortality attributed to tobacco use, to indicate the principal finding and methodological differences existing among them.

## Methods

In order to obtain papers that addressed the methodology employed for attributing mortality to tobacco use, a review of the MEDLINE electronic databases was carried out in January 2013 for the period 1998 to 2005. Search terms were used, including “attributable risk”, “mortality”, “smoking”, in combination with the key words “tobacco”. The search was completed with a manual review of the bibliographic references cited by the papers retrieved and of other publications, such as the monographs published by the Centers for Disease Control and Prevention (CDC) concerning data about SAM in Canada, USA and Portugal.

The main inclusion criterion was the use of modelling to estimate attributable mortality. Papers merely describing mortality, such as cohort follow-up or mortality studies were excluded. Articles published in English, were included; those in other languages were excluded irrespective of whether they contained a summary in English. No restriction was taken into account on location of publication or period of the study.

A total of 186 articles were found, 30 of which were selected since they used modelling methods to estimate tobacco attributable risk. Eighteen papers were excluded since they used an age range < 25 years. As a result, 12 articles published between 1998 and 2005 were included in the analysis.

The following items were extracted from the chosen articles: authors, location where the study was carried out, publication year, age range of the population under study, way of calculating the SAM, main findings and limitations or problems identified.

## Results and discussion

### Type of models and used methods

Smoking attributable mortality (SAM) has been used in studies in the form SAM%, giving the proportion of all deaths in general or of those with a specific cause that are attributable to tobacco. The applied methodology in these studies can be classified under three categories: prevalence-based analysis in cohort studies (SAMMEC method), prevalence-based analysis in case–control studies, smoking impact ratio method. The methods differ in terms of calculation processes, information requirement, data sources and assumptions required for their application. Table 1 shows the results of the articles, by year, location of publication, method of calculating the SAM and age range under analysis. The main characteristics of the different indirect methods are described below.

**Table 1:** Methods used in calculating the Smoking Attributable Mortality (SAM) by location and date of study

**Ref**	**Location/ Year of study**	**Method used**	**Age range**	**Source of data**
[21]	Canada, 2001	SAMMEC	>35	Canadian community health survey;
				Mortality registry
[22]	Brezil, 2003	SAMMEC	>35	Brazilian mortality system;
				Household survey on NCD risk factors
[23]	Israel 2003	SAMMEC	>35	National smoking prevalence data;
				National mortality data survey
[24]	Portugal 2005	SAMMEC	>35	National heath enquiries
[25]	USA, 2005	SAMMEC	>35	National center for health statistics’ and US mortality data
[26]	Taiwan, 2001	SAMMEC	>30	National health interview survey;
				Official projection of number of deaths
[35]	China, 1998	Case	>40	Official death certificates;
				Disease surveillance points
[33]	India 2003	Case	>25	Case control survey among mal
[34]	Hong kong 2004	Case	>35	Death registry
[37]	South Africa 2004	Case	>35	The South African death notification system
[40]	World 2003	SIR	>30	WHO GB database
[13]	World 2005	SIR	>30	WHO GB database cardiovascular

#### Prevalence-based analysis in cohort studies (SAMMEC)

This method is the most commonly employed in the literature [19-26]. Attributable deaths are calculated for each cause of mortality using the following formula: AM = OM × PAF; where AM is the mortality attributed to a specific factor (SAM in the case of smoking), OM the observed mortality, and PAF the population attributable fraction. The first step consists in selecting the most relevant smoking related pathologies for which reliable data are available. These pathologies and their codification in two International Disease Classifications, ICD 9, Clinical Revised Modification, and ICD 10, are listed in Table 2[27]. The second step establishes the quantitative relationship between smoking and the selected pathologies.

**Table 2:** ICD 9-CM and ICD 10 codes for smoking related diseases

**Malignant cancers**	**ICD 9 - CM**	**ICD 10**	
Trachea, lungs, bronchi	162	C33 – C34	
Esophagus	150	C15	
Stomach	151	C16	
Pancreas	157	C25	
Larynx	161	C32	
Lips, oral cavity, pharynx	140-149	C00 – C14	
Neck of the uterus	180	C53	
Kidney, renal pelvis	189.1	C64-C65	
Urinary bladder	188	C67	
Acute myeloid leukaemia	205	C92.0	
**Cardiovascular diseases**			
Ischemic heart disease			
<65 years	410-414	I20-I25	
>65 years			
Cerebrovascular disease			
<65 years	433-434	I63 – I69	
>65 years	436-438		
Atherosclerosis	440	I 70	
Aortic anevrysm	171.9	I71	
Other arterial disease	440-448	I72-I78	
Other cardiac diseases	412-414	I25	
**Respiratory diseases**			
Bronchitis, emphysema	490-492	J40-J43	
Chronic airway obstruction	496	J44 – J46	
Pneumonia, influenza	480-487	J10-J18	

In order to do that, we used epidemiological concepts of relative risk and population attributable risk. Relative risk (RR) is the ratio between the risk of a disease or death for a population exposed to smoking and this risk for a non-exposed population. To calculate PAF, different methods exist [28], the most widely method used is based on the formula proposed by Levin [29] which divides the population into various categories according to tobacco use (non-smokers, ex-smokers and smokers):

$$\begin{array}{ll} \mathrm{PAF} & =\left(\left(p0+p1\times \mathrm{RR}1+p2\times \mathrm{RR}2\right)-1\right)\\ & \;/\left(p0+p1\times \mathrm{RR}1+p2\times \mathrm{RR}2\right); \end{array}$$

where p0, p1 and p2 represent the prevalence of non-smokers, smokers and ex-smokers, respectively. Data are drawn from registries in the case of observed mortality and from surveys in the case of smoking prevalence. RR1 and RR2 refer to the risk of dying for smoking related pathologies of smokers and ex-smokers respectively compared to a baseline population of non-smokers. The relative risks (RRs) employed in the calculations are extracted mainly from the prospective cohort study conducted by the American Cancer Society, i.e., the Cancer Prevention Study II (CPS II) [30,31]. PAF is estimated for the 22 tobacco related diseases [32]. The CDC’s SAMMEC (Smoking-Attributable Mortality, Morbidity, and Economic Cost) computer software application [32] uses this methodology. SAMMEC is a software package commonly used in the United States to estimate attributable mortality due to smoking, years of potential life lost and indirect mortality costs.

#### Prevalence-based analysis in case–control studies

Employing a similar calculation procedure to the previous method, this one emerged as a consequence of the objections raised by certain researchers about using RRs to estimate smoking attributable mortality from other countries [33,34]. This method has been used to estimate mortality attributable to tobacco use in China when the epidemic was still in the initial phase [35,36] and South Africa [37]. To apply this method, it is necessary to know the total deaths for all causes among subjects aged 35 years or more for a given period of time.

By interviewing survivors, information is collected retrospectively on smoking habits of deceased subjects 15 years before their death. Based on a case–control study risks are estimated. Once these risks obtained, the population attributable fraction (PAF) can then be calculated, applying the formula:

$$\mathrm{PAF}=P\times \left(1-\left(1/\mathrm{RR}\right)\right);$$

where P is the proportion of deaths occurring among smokers and RR the relative risk calculated as OR after completion of a case–control study. When the PAF has been calculated, deaths attributed to tobacco use (AM) in the study population can be estimated as follows:

AM=OM×PAF.

#### Smoking impact ratio method

To capture the accumulated hazard of smoking, the SIR-method (smoking impact ratio) was elaborated by Peto et al., that is adapted to the specific conditions of developing countries [38-40]. The method uses lung cancer mortality data, which are available or can be estimated using various methods, as an indirect indicator of the accumulated hazards of tobacco smoking. Background-adjusted SIR is defined as population lung cancer mortality in excess of never-smokers relative to excess lung cancer mortality for a known reference group of smokers, adjusted to account for differences in never-smoker lung cancer mortality rates across populations [41,13].

This model may estimate mortality independently of the prevalence of smoking in the study population. To apply this method, one needs to know the age- and sex-specific lung cancer mortality rates in the target country (CLC) and also in never-smokers of the same population (NLC), the relative risks for all diseases and disorders causally related to tobacco, except lung cancer; and the cause-specific lung cancer mortality rates in smokers (S*LC) and never smokers (N*LC), taken from a cohort study. Peto et al. used data drawn from the CPS II. SIR is calculated by age and sex. Age groups were 30–44, 45–59, 60–69, 70–79 and ≥80. No deaths before the age of 30 were attributed to smoking because there are few cancer deaths before 30 and RRs are unstable.

The second step of this process consists of computing the population etiological fraction (PEF) on the basis of the previously calculated summarized prevalence (SIR) and the relative risks of dying due to the respective causes (RR), by age group and sexx as per the CPS II.

$$\mathrm{PEF}=\mathrm{SIR}\;\left(\mathrm{RR}-1\right)/(1+\left(\mathrm{SIR}\left(\mathrm{RR}-1\right)\right).$$

The last step in this procedure would involve applying the following formula: *AM* = *OM* × *PEF*, in order to obtain the estimation of attributed mortality, AM, in accordance with the PEF previously calculated and the observed mortality, OM.

### Smoking attributable mortality

Table 3 presents the principle findings and general characteristics of the studies. The studies were organized according to the methods used for calculating the SAM. Most studies used the relative risk (RR) from CPS II. Smoking related pediatric illnesses, deaths caused by to passive smoking and tobacco related fires were not included in the most studies analyzed here with some exceptions [42]. Most studies using prevalence based methods used an age range >35 years for the calculation of the SAM, with some exceptions [26,33,36]. Studies using Peto’s method used age rage >30 years.

**Table 3:** Summary of Smoking Attributable Mortality (SAM) as reported in the articles included in the review

	**SAM in thousands of deaths (proportion of total adult mortality)**			**Comments**
**Ref**	**Male**	**Female**	**Both**	
[19]	27.600 (26.2%)	13.170 (13.4%)	40.770 (20.0%)	SAM as a rate of total death;
[22]	16.896 (18.1%)	7.326 (8.1%)	24.222 (13.6%)	Mortality estimation according to smoking related disease group in 16 Brazilians cities
[23]	3.680	2595	6.275 (16.3%)	Use of the lagged SAMMEC models
[24]	9.890 (17.8%)	2.725 (5.2%)	12.615 (11.7%)	Estimation of other tobacco burdens (Dalys, deaths…)
[25]	259.5	178.5	438	Estimation of annual deaths between1997 and 2001, inclusion of second hand smoking death
[26]	16.123 (22.2%)	2.680 (5.9%)	18.803 (16.0%)	Take into consideration induction time.
[32]	500 (13%)	100 (3%)	600 (12%)	Estimation of future burden under scenarios of health promotion
[33]	-	-	550	Estimation of men deaths only
[36]	2.534 33%	0.169 (5%)	2701 (25%)	Graphic form of data
[34]	-	-	20000 (8%)	Deaths estimation by cause: lung cancer, COPD, tuberculosis and vascular deaths.
[38]	2410 (14%)	380 (3%)	2020 (9%)	Assumes constant worldwide lung cancer mortality rates among never smokers.
				Worldwide use.
[13]	-	-	670 (7%)	Estimation of cardio vascular deaths

Ezzati and Lopez showed that the general rate of SAM globally was 12%; 18% among men and 5% among women. Lung cancer was the disease with the highest fraction attributable to smoking. 71% of all lung cancers or 0 · 85 million deaths (79% or 0 · 69 million deaths among men and 48% or 0 · 16 million deaths among women) were attributable to smoking. In the year 2000, 11% of total global cardiovascular deaths, were due to smoking; 17% for men and 5% for women [39]. SAM represented 9% of total adult mortality in developing countries where it represented 14% of total mortality in adult men (2 · 02 million deaths) and 3% of total mortality in women (380 000 deaths). In these countries, SAM represented 65% of lung cancer deaths in men aged between 30 and 69 years and caused 7% of global cardiovascular death (670 000 deaths) [13]. In the studies assessed here, the general SAM was between 8% in Southern Africa and 25% in Hong Kong; in Brazil, it represents 13.6% of total deaths [34,24,22]. In men, the rate was between 13% in China, 18.1% in Brazil and 33% in Hong Kong and in women between 3% in China, 8.7% in Brazil and 13.4% in Canada. In Southern Africa, 58% of lung cancer mortality was attributable to tobacco (61% in males and 48% in females). Smoking caused 18% of ischemic heart diseases death. Smoking caused 30.2% and 29.1% of cancer mortality respectively in Brazil and Israel. It is responsible for 32.4% and 42.6% of cardiovascular mortality in the same countries [22,23].

### Discussion

Our review shows that mortality from smoking varied greatly among different countries (from 8% in South Africa to 25% in Hong Kong). SAM is highly concentrated among men (33% in Hong Kong and 22.2% in Taiwan) [34,26]. A comparison between the results that Ezzati and Lopez [40] and Ezzati et al. [41] reported for SAM% with those from studies that use a more uniform methodology [25] shows that the general mortality rate (18% - 23%) was higher for the world and for developed countries in the former studies.

Although it is very difficult to generalize about developing countries as there are huge variations in the smoking epidemic determined by diverse demographics and economic and cultural determinants. First, mortality attributable to smoking in these regions is highly concentrated among men (84% of smoking-attributable deaths). Smoking killed three times as many men as women in industrialized countries and almost seven times as many in developing countries. Second, compared with industrialized countries, developing countries have a higher proportion of SAM at age 30 to 69 (62% in less-developed countries, compared with 49% in industrialized nations) [41]. The tobacco related illnesses that most contribute towards the SAM in developing countries were cancer of the trachea/ bronchial/ lungs, ischemic heart disease, COPD and cerebrovascular diseases [26,36]. Ezzati and Lopez also found cardiovascular disease, COPD and lung cancer to be the three principal causes of smoking related deaths in developed and developing countries in the year 2000 [42].

The first limitation affecting comparison of the cited studies stems from the use of a different methodological approach in the various studies. The studies reviewed here are quite heterogeneous in many aspects: the method for calculating the attributable fraction, the inclusion or not of certain tobacco-related diseases in adults or children, the age range considered, the inclusion of death by burning, passive smoking and the application of the current prevalence to calculate the SAM. All these factors influence the results of the attributable mortality. The second limitation resides in the absence of a universal definition of the categorization of tobacco use. To view smokers as a single entity could lead to a distorted mortality estimate, since failure to take account of the number of cigarettes smoked, age at initiation and years of smoking [42,43].

The third limitation, present mainly in the prevalence based methods, centers on their reliance on current smoking prevalence’s to reflect mortality occasioned by tobacco use in previous years. Knowing current smoking prevalence could be a great help when it comes to predicting future mortality, it might fail however with respect to the present mortality [43]. The use of current prevalence may overestimate or underestimate the attributable mortality. In countries where the prevalence is decreasing, the use of current prevalence is conservative in the proportional attribution method. In Israel, where tobacco use has been decreasing for several decades, using a lagged approach – which took into account the fact that smoking rates between 1959 and 2003 contribute to SAM in 2003, due to lag factors - produced a SAM overestimate of 50% on the SAMMEC categories [23].The opposite in countries where prevalence is increasing. As yet, this problem has no easy solution, due to the absence of historical series of smoking prevalence in most countries.

Peto et al. avoided the problem entailed in prevalence dependent methods of attribution. For the application of their estimation procedure, lacks of knowledge of the tobacco consumption or latency and induction periods are no limitations. Smoking impact ratio method defined synthetic prevalence as an indicator that summarizes a population’s smoking history, and calculate it by assuming CPS II data on lung cancer mortality rates among smokers and non-smokers to be valid. The use of these two sets of data gave rise to numerous criticisms especially representativeness of the CPS population. Most of the population included in this cohort study was middle class, which may result in lung cancer mortality in non-smokers being underestimated, which in turn may lead to an overestimation of lung cancer mortality attributable to tobacco use and, by extension, to an overestimation of the summarized prevalence [44-46].

Possibilities of confounding are also not properly taken into account by the fact that smoker/nonsmoker relative risks for diseases other than lung cancer are estimated from unadjusted CPS II data and assumed to apply to countries with a very different distribution of risk factor exposure than the CPS II population, a population which is not even representative of the United States. The method implausibly assumes that lung cancer rates in lifelong nonsmokers do not vary by country and by year, thus ignoring possible diseases are unlikely to be representative, in terms of effects of other risk factors [47].

The fourth limitation centers on the absence of world-wide risk indicators that would reflect the degree of association between tobacco and smoking related-causes of mortality. Although drawn from different sources, the RRs used in the various studies mainly came from the CPS II [30,31]. Applying these risks to populations other than that of the USA aroused criticism. A solution to these problems was sought through a re-analysis of the data [48,49], and the RRs were shown robust. The absence of a simulation study involving and comparing all calculations procedures do not allow us to recommend a method over other one. Data availability should be taken into account when choosing a method. These types of methods furnish estimates that constitute valuable information and help forming a more accurate picture of the problem that smoking poses to world health.

## Conclusion

This analysis of different studies has shown the powerful impact that tobacco consumption has on the mortality of populations. Many low- and middle-income countries are still in early stages of the tobacco epidemic. In these nations, smoking attributable mortality is low compared to developed countries. In view of the expected demographic and epidemiological transitions and of current smoking patterns in these counties, the health loss due to smoking will grow even larger unless effective interventions and policies that reduce smoking among men and prevent increases among women are implemented.

## Competing interest

The authors declare that they have no competing interests.

## Authors’ contributions

NT performed protocol and wrote the manuscript. CR participated in writing the manuscript. MO participated in writing. CN performed protocol and wrote the manuscript. All authors read and approved the final manuscript.
